# Genome-Wide Identification of Direct Targets of the TTG1–bHLH–MYB Complex in Regulating Trichome Formation and Flavonoid Accumulation in *Arabidopsis Thaliana*

**DOI:** 10.3390/ijms20205014

**Published:** 2019-10-10

**Authors:** Zelou Wei, Yalong Cheng, Chenchen Zhou, Dong Li, Xin Gao, Shuoxin Zhang, Mingxun Chen

**Affiliations:** 1 State Key Laboratory of Crop Stress Biology for Arid Areas and College of Agronomy, Northwest A&F University, Yangling 712100, Shaanxi, China; weizelou@nwafu.edu.cn (Z.W.); zhouchenchen@nwafu.edu.cn (C.Z.); ld@nwafu.edu.cn (D.L.); bestgaoxin@nwsuaf.edu.cn (X.G.); 2 College of Forestry, Northwest A&F University, Yangling 712100, Shaanxi, China; chengyalong@nwsuaf.edu.cn; 3 Qinling National Forest Ecosystem Research Station, Huoditang, Ningshan 711600, Shaanxi, China

**Keywords:** MBW complex, TTG1, trichome formation, flavonoid accumulation, *Arabidopsis thaliana*

## Abstract

Extensive studies have shown that the MBW complex consisting of three kinds of regulatory proteins, MYB and basic helix–loop–helix (bHLH) transcription factors and a WD40 repeat protein, TRANSPARENT TESTA GLABRA1 (TTG1), acts in concert to promote trichome formation and flavonoid accumulation in *Arabidopsis thaliana*. TTG1 functions as an essential activator in these two biological processes. However, direct downstream targets of the TTG1-dependent MBW complex have not yet been obtained in the two biological processes at the genome-wide level in *A. thaliana*. In the present study, we found, through RNA sequencing and quantitative real-time PCR analysis, that a great number of regulatory and structural genes involved in both trichome formation and flavonoid accumulation are significantly downregulated in the young shoots and expanding true leaves of *ttg1-13* plants. Post-translational activation of a TTG1-glucocorticoid receptor fusion protein and chromatin immunoprecipitation assays demonstrated that these downregulated genes are directly or indirectly targeted by the TTG1-dependent MBW complex in vivo during trichome formation and flavonoid accumulation. These findings further extend our understanding of the role of TTG1-dependent MBW complex in the regulation of trichome formation and flavonoid accumulation in *A. thaliana*.

## 1. Introduction

TRANSPARENT TESTA GLABRA1 (TTG1) encodes a WD40 repeat transcription factor that plays pleiotropic roles in the regulation of seed development and postembryonic processes in *Arabidopsis thaliana* [1,2,3,4,5,6,7,8]. During seed development, it not only promotes the biosynthesis of proanthocyanidins (PAs) [1,2,3,8,9] but also accelerates the production of mucilage and columella [1,8,10,11]. By contrast, it negatively regulates the accumulation of seed storage reserves, including fatty acids and proteins during seed maturation [7,8]. In the postembryonic process, TTG1 functions in root development including root length and hairs [12,13] and the response to abiotic stresses [13,14]. Further, TTG1 also acts as a key transcriptional activator in trichome formation [1,3,12,15] and flavonoid deposition [1,2,3,9,16], and the TTG1-dependent regulatory network in the two biological processes has been extensively studied in *A. thaliana*.

Trichomes are single-celled and hairy structures that develop into an epidermis of the aerial parts, including leaves, stems, and sepals, in *A. thaliana*. They are related with water regulation, temperature control, and protection against biotic and abiotic stresses [15,17,18,19]. Trichome formation is activated by the MYB–basic helix–loop–helix (bHLH)–WD40 (MBW) complex, which includes an R2R3-type MYB-related transcription factor (GLABRA1 (GL1) or MYB23), a bHLH protein (GLABRA3 (GL3) or ENHANCER OF GLABRA3 (EGL3)), and the WD40-repeat protein TTG1 [12,15,20,21]. The GL1-GL3/EGL3–TTG1 complex promotes trichome formation through inducing the expression of *GLABRA2* (*GL2*) and some single-repeat R3 MYB transcription factors, and then the induced MYB transcription factors in turn impede the complex formation by competing with GL1 for binding GL3 or EGL3 [22,23,24]. TTG1 directly induces the *TRANSPARENT TESTA GLABRA2* (*TTG2*) expression [25]. The further study indicated that TTG1 represses the activation of the *CAPRICE* (*CPC*) promoter by GL1 and GL3, and GL1 suppresses the activation of the *TRIPTYCHON* promoter by GL3 and TTG1 [26].

Flavonoids, as secondary metabolites, are ubiquitously produced in higher plants and can be categorized into three major classes in *A. thaliana*, namely, flavonols, PAs, and anthocyanins [9,27,28]. They are involved in feeding and pollination attraction, rhizosphere signaling, auxin movement, nutrient retrieval during senescence, and protection against phytopathogens and sunlight irradiance [29,30,31,32,33,34,35]. Further, flavonoids also serve as the source of beneficial micronutrients for humans, useful for human health and protecting against many diseases [36,37,38,39,40]. The MBW complex, which promotes flavonoid accumulation, consists of an R2R3 MYB gene (PRODUCTION OF ANTHOCYANIN PIGMENT 1 (PAP1), PAP2, MYB113 or MYB114), a bHLH transcription factor (TRANSPARENT TESTA 8 (TT8), GL3 or EGL3), and the WD40-repeat protein TTG1 [41,42,43,44,45]. TTG1 directly activates *BANYULS* (*BAN*) expression through TT8 stability in *A. thaliana* siliques [46].

Such studies as the abovementioned indicate the TTG1-dependent MBW complex acts as a regulatory hub in the modulation of trichome formation and flavonoid accumulation, and TTG1 functions as an essential activator in the two biological processes, in *A. thaliana*. However, direct downstream targets of the TTG1-dependent MBW complex have so far not yet been obtained at the genome-wide level in *A. thaliana*.

In the present study, we found that a great number of regulatory and structural genes involved in trichome formation and flavonoid accumulation are significantly downregulated in the young shoots and expanding true leaves of *ttg1-13* plants through transcriptome analysis. We demonstrated that these downregulated genes are directly or indirectly targeted by the MBW complex in vivo, using the approaches of the dexamethasone (DEX)-inducible gene expression system and chromatin immunoprecipitation (ChIP), during trichome formation and flavonoid accumulation in *A. thaliana*.

## 2. Results

### 2.1. Genome-Wide Analysis of Downstream Targets Involved in Trichome Formation and Flavonoid Accumulation in ttg1-13 Young Shoots and Expanding True Leaves

The loss-of-function mutant of *ttg1-13* (CS67772) as a mixed Columbia/L*er* background was obtained by fast neutron mutagenesis from the Arabidopsis Biological Resource Center (ABRC). This mutant was backcrossed twice to the wild type Col-0 to purify its background and eliminate other possible mutations [14], and then used in this study. There are no trichome formation and flavonoid deposition in *ttg1-13* young shoots and true leaves (Figure S1, [14]), which is consistent with the previous evidence that TTG1 positively promotes trichome formation and flavonoid accumulation in *A. thaliana* [1,2,3,9,12,15,16]. Trichomes are commonly present in the surface of stems and leaves, and anthocyanins, the most conspicuous class of flavonoids, are frequently produced in young shoots and expanding leaves [47]. Further, TTG1 is highly expressed in young shoots and expanding true leaves [25,46]. Therefore, to identify downstream targeted genes of the TTG1-dependent MBW complex involved in trichome formation and flavonoid accumulation at the genome-wide level, the tissues of young shoots and expanding true leaves from the wild type and *ttg1-13* plants at 20 days after germination (DAG) (Figure S1) were used for the RNA-seq experiment.

The RNA-seq analysis identified 987 differential expressed genes (DEGs) (Tables S1 and S2), of which 732 were expressed at lower levels (‘downregulated’; Table S1) and 255 at higher levels (‘upregulated’; Table S2) in the *ttg1-13* young shoots and expanding true leaves. Based on the functional annotations, 14 and 15 of the downregulated genes were found to be involved in trichome formation and flavonoid biosynthesis, respectively, but none of the upregulated genes were involved in the two biological processes (Table 1 and Table 2; Tables S1 and S2). We noted that the number of downregulated genes related to the primary metabolic processes, such as carbohydrates, amino acids and proteins, and lipids, was more than that of the upregulated genes, as was the number of other several biological processes, including cell wall, signaling transduction, oxidation–reduction, and stress/defense response, in the *ttg1-13* young shoots and expanding true leaves (Tables S1 and S2). These likely indicated that the TTG1-dependent MBW complex functions as a key transcriptional activator in the regulation of trichome formation, flavonoid biosynthesis, and other major biological processes.

### 2.2. Validation of Downstream Targets Related to Trichome Formation and Flavonoid Accumulation in ttg1-13 Young Shoots and Expanding True Leaves

**Figure 1:**
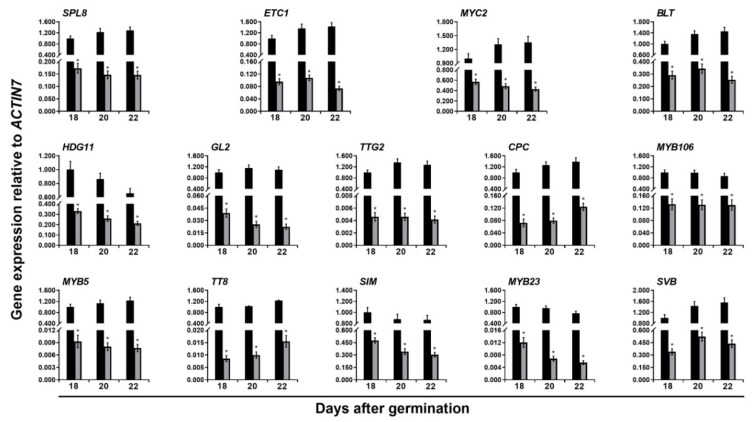
Dynamic expression analysis of differentially expressed genes involved in trichome formation in the young shoots and expanding true leaves between the wild type (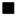) and *ttg1-13* (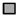) plants at 18, 20, and 22 days after germination. The house-keeping gene *ACTIN7* was used as the internal control. The expression of each gene was first calculated relative to *ACTIN7* and then normalized to its expression level at 18 days after germination in the wild type that was set to 1. Asterisks indicate significant differences in gene expression compared with the wild type control (two-tailed paired Student’s *t* test, *p* ≤ 0.05). Values are means ± SD (*n* = 3). Error bars denote SD.

**Figure 2:**
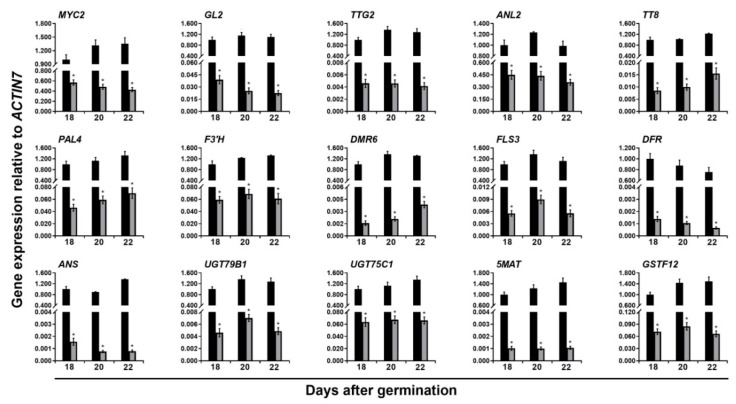
Dynamic expression analysis of downregulated genes involved in flavonoid biosynthesis in the young shoots and expanding true leaves between the wild type (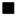) and (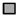) *ttg1-13* plants at 18, 20, and 22 days after germination. The house-keeping gene *ACTIN7* was used as the internal control. The expression of each gene was first calculated relative to *ACTIN7* and then normalized to its expression level at 18 days after germination in the wild type that was set to 1. Asterisks indicate significant differences in gene expression compared with the wild type control (two-tailed paired Student’s *t* test, *p* ≤ 0.05). Values are means ± SD (*n* = 3). Error bars denote SD.

To further confirm the expression of these downregulated genes related to trichome formation and flavonoid accumulation, quantitative real-time PCR (qRT-PCR) was then used to analyze their dynamic expression levels in the *ttg1-13* young shoots and expanding true leaves at 18, 20, and 22 DAG. The results showed that the expression levels of all these genes involved in trichome formation and flavonoid accumulation are significantly reduced at 20 DAG, which is highly consistent with the RNA-seq results (Figure 1 and Figure 2; Table 1 and Table 2, Tables S1 and S2). For the trichome-inducing genes, the expression levels of 9 regulatory genes, *SQUAMOSA PROMOTER BINDING PROTEIN–LIKE 8* (*SPL8*), *ENHANCER OF TRYAND CPC 1* (*ETC1*), *BRANCHLESS TRICHOMES* (*BLT*), *HOMEODOMAIN GLABROUS11* (*HDG11*), *CPC*, *MYB106*, *MYB5*, *SIAMESE* (*SIM*), and *MYB23*, and 1 structural gene, *SMALLER WITH VARIABLE BRANCHES* (*SVB*), were always significantly lower in the *ttg1-13* young shoots and expanding true leaves relative to the wild type plants at 18 and 22 DAG (Figure 1; Table 1 and Table S1). On the other hand, the expression levels of 11 genes related to flavonoid biosynthesis were also greatly downregulated in the *ttg1-13* young shoots and expanding true leaves at 18 and 22 DAG, which includes 1 regulatory gene, *ANTHOCYANINLESS2* (*ANL2*), and 10 structural genes in the flavonoid biosynthetic pathway, *PHENYLALANINE AMMONIA-LYASE4* (*PAL4*), *FLAVONOID 3’-HYDROXYLASE* (*F3’H*), *DOWNY MILDEW RESISTANT 6* (*DMR6*), *FLAVONOL SYNTHASE3* (*FLS3*), *DIHYDROFLAVONOL REDUCTASE* (*DFR*), *ANTHOCYANIDIN SYNTHASE* (*ANS*), *ANTHOCYANIN 3-O-GLUCOSIDE: 2’’-O-XYLOSYLTRANSFERASE* (*UGT79B1*), *ANTHOCYANIN 5-O-GLUCOSYLTRANSFERASE* (*UGT75C1*), *ANTHOCYANIN* 5-*O*-GLUCOSIDE-6’’-*O*-MALONYLTRANSFERASE (*5MAT*), and *GLUTATHIONE S-TRANSFERASE F12* (*GSTF12*) (Figure 2; Table 2 and Table S1). It is notable that 4 regulatory genes involved in both trichome formation and flavonoid accumulation, namely *MYC2*, *GL2*, *TTG2*, and *TT8*, were dramatically downregulated in the *ttg1-13* young shoots and expanding true leaves at 18 and 22 DAG (Figure 1 and Figure 2; Table 1 and Table 2, and Table S1). Previous studies have demonstrated that *TTG2*, *TT8*, D*FR*, *F3’H*, and *UGT79B1* are targets of TTG1 [25,44,69], which is a proof of our successful gene expression analysis in this study. Taken together, these results further indicated that the TTG1-dependent MBW complex promotes trichome formation and flavonoid accumulation by activating a series of regulatory and structural genes involved in trichome development and flavonoid biosynthesis, respectively, in *A. thaliana* young shoots and expanding true leaves.

### 2.3. Identification of Direct Downstream Targets Contributing to Trichome Formation and Flavonoid Accumulation Regulated by the TTG1-Dependent MBW Complex in Young Shoots and Expanding True Leaves

To investigate how the TTG1-dependent MBW complex controls the mRNA expression of downstream targeted genes, we created a steroid-inducible version of TTG1 in the background of *ttg1-13*, in which the *TTG1* gene was fused to the rat glucocorticoid receptor (GR) and driven by the 35S promoter. We isolated a *ttg1-13 35S:TTG1-GR* transgenic line, which fully rescued the phenotypes of *ttg1-13* leaves without trichomes and flavonoids after DEX treatment every other day after germination (Figure S2), whereas the mock-treated *ttg1-13 35S:TTG1-GR* exhibited similar trichome and flavonoid phenotypes in leaves with *ttg1-13* (Figures S1 and S2). This indicated that the TTG1-GR fusion protein has a biological function like that of the wild type TTG1 upon steroid induction.

**Figure 3:**
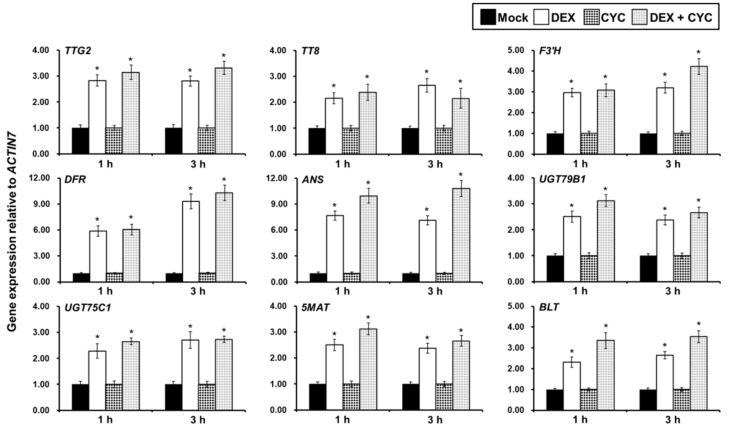
Induced TRANSPARENT TESTA GLABRA1 (TTG1) activity transcriptionally promotes the expression of several genes involved in flavonoid biosynthesis, including *TTG2*, *TT8*, *F3’H*, *DFR*, *ANS*, *UGT79B1*, *UGT75C1*, and *5MAT*, and the gene related to trichome formation *BLT* in the young shoots and expanding true leaves. The *ttg1-13 35S:TTG1-GR* young shoots and expanding true leaves at 20 days after germination were mock-treated (Mock) or treated with 10 μM dexamethasone (DEX), 5 μM cycloheximide (CYC), or 10 μM DEX plus 5 μM CYC (DEX + CYC). The expression of these genes was determined after 1 or 3 h of treatment using qRT-PCR analyses. The house-keeping gene *ACTIN7* was used as the internal control. The expression level of each gene was first calculated relative to *ACTIN7*, and its expression levels in both Mock and CYC treatments were set to 1. Asterisks indicate significant differences in gene expression in DEX-treated samples compared with their respective controls (two-tailed paired Student’s *t* test, *p* ≤ 0.05). Values are means ± SD (*n* = 3). Error bars denote SD.

By utilizing the established steroid-inducible activation of TTG1, we further detected whether the expression of these downregulated genes is activated by TTG1 activity (Figure 1 and Figure 2). DEX treatment of *ttg1-13 35S:TTG1-GR* young shoots and expanding true leaves at 20 DAG for 1 or 3 h resulted in a significant increase in the expression of *TTG2*, *TT8*, *F3’H*, *DFR*, *ANS*, *UGT79B1*, *UGT75C1*, *5MAT*, *BLT*, *ANL2*, *PAL4*, *DMR6*, *FLS3*, *GSTF12*, *MYB5*, and *MYB23*, compared with that of the mock-treated controls (Figure 3 and Figure S3). To determine whether 35S:TTG1-GR directly or indirectly represses these genes, we repeated DEX applications to the 35S:TTG1-GR young shoots and expanding true leaves of the *ttg1-13* background in the presence of the protein synthesis inhibitor cycloheximide (CYC), because the induction of TTG1-GR activity by DEX does not require protein synthesis. We found that the combined treatment of DEX and CYC for 1 or 3 h only dramatically promoted the expression of *TTG2*, *TT8*, *F3’H*, *DFR*, *ANS*, *UGT79B1*, *UGT75C1*, *5MAT*, and *BLT* (Figure 3 and Figure S3). These indicated that these 9 genes are immediate targets transcriptionally induced by the TTG1-dependent MBW complex in the young shoots and expanding true leaves (Figure 3), whereas the activation of the other 12 genes in the *ttg1-13* mutant is reliable on other intermediate proteins (Figure S3).

**Figure 4:**
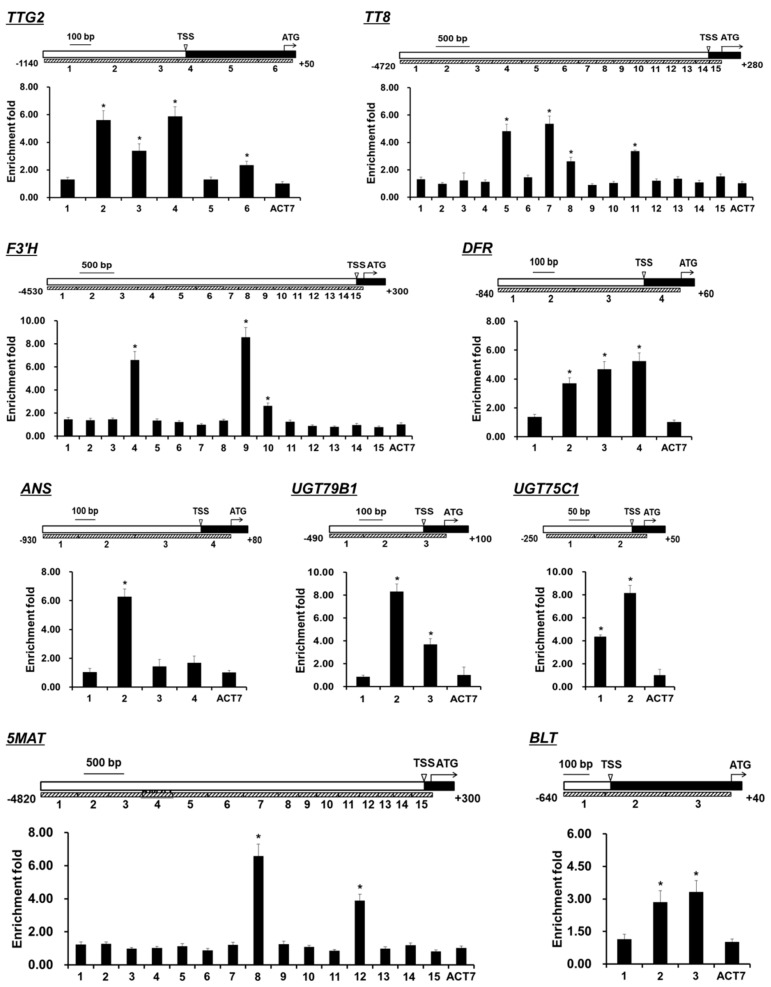
Schematic diagrams illustrate the promoter regions of *TTG2*, *TT8*, *F3’H*, *DFR*, *ANS*, *UGT79B1*, *UGT75C1*, *5MAT*, and *BLT*, and ChIP analysis indicates the TTG1-dependent MBW complex binding to their promoter regions in in the young shoots and expanding true leaves at 20 days after germination. The transcriptional start site (TSS) and exon are indicated by black boxes, whereas promoter regions are indicated by white boxes. Gray boxes represent the DNA fragments amplified in ChIP analysis for each gene. The enrichment fold of each fragment was calculated first by normalizing the amount of a target DNA fragment against a genomic fragment of *EF1aA4* as an internal control and then by normalizing the value for dexamethasone-treated *ttg1-13 35S:TTG1-GR* against that for mock-treated *ttg1-13 35S:TTG1-GR*. An ACTIN7 (ACT7) fragment was amplified as a negative control. Asterisks indicate significant differences in comparison with the enrichment of the ACT7 fragment (two-tailed paired Student’s *t* test, *p* ≤ 0.05). Values are means ± SD (*n* = 3). Error bars denote SD.

To explore whether the TTG1-dependent MBW complex binds directly to the promoter regions of *TTG2*, *TT8*, *F3’H*, *DFR*, *ANS*, *UGT79B1*, *UGT75C1*, *5MAT*, and *BLT* to regulate their expressions, the young shoots and expanding true leaves from mock- or DEX-treated *ttg1-13 35S:TTG1-GR* plants with were used for the ChIP assay. To cover all the possible *cis*-elements, sufficient pairs of primers were designed in the promoter regions of these 9 genes (Figure 4). The ChIP results showed that TTG1-GR was associated with the promoter regions near fragments 2, 3, and 4 of *TTG2*, fragments 5, 7, 8, and 11 of *TT8*, fragments 4, 9, and 10 of *F3’H*, fragments 2, 3, and 4 of *DFR*, fragment 2 of *ANS*, fragments 2 and 3 of *UGT79B1*, fragments 1 and 2 of *UGT75C1*, fragments 8 and 12 of *5MAT*, and fragments 2 and 3 of *BLT* (Figure 4). These results collectively suggested that the TTG1-dependent MBW complex binds directly to the loci of *TTG2*, *TT8*, *F3’H*, *DFR*, *ANS*, *UGT79B1*, *UGT75C1*, *5MAT*, and *BLT* to promote their expression.

## 3. Discussion

Transcriptional regulation is considered to be the essential mechanism controlling the gene expression of metabolic pathways in higher plants. Extensive studies have elucidated the TTG1-dependent MBW complex that controls trichome formation and flavonoid accumulation in *A. thaliana*. However, its direct targeted genes are still largely unknown in the regulation of trichome formation and flavonoid accumulation in *A. thaliana*. In this study, we identified a series of new downstream targeted genes for trichome formation and flavonoid accumulation directly or indirectly regulated by the TTG1-dependent MBW complex at the genome-wide level in *A. thaliana* young shoots and expanding true leaves.

We found that there were 4 downregulated regulatory genes involved in both trichome formation and flavonoid biosynthesis, *TTG2*, *TT8*, *GL2*, and *MYC2* (Figure 1 and Figure 2; Table 1 and Table 2), among which the former two genes and the latter two genes were directly and indirectly regulated by the TTG1-dependent MBW complex (Figure 3 and Figure 4, and Figure S3), in the *ttg1-13* young shoots and expanding true leaves. The TTG2 protein encoding a WRKY transcription factor plays positive roles in trichome initiation and flavonoid accumulation and is also directly induced by TTG1 [25,57], which is consistent with our results (Figure 3 and Figure 4). The TT8 transcription factor is essential for trichome initiation in the margin of true leaves [62] and positively regulates flavonoid biosynthesis [68,69]. GL2 functions as a transcriptional activator and repressor in trichome initiation [22,23,24] and anthocyanin biosynthesis [66], respectively. The previous study showed that GL2 inhibits anthocyanin biosynthesis by directly repressing the expression of *PAP1*, *PAP2*, *MYB113*, *MYB114*, and *TT8* [66], which theoretically should be increased because of the lower expression of *GL2* in the *ttg1-13* young shoots and expanding true leaves. However, the *TT8* expression was significantly reduced (Figure 1 and Figure 2; Table 1 and Table 2, and Table S1), and the expression of *PAP1*, *PAP2*, *MYB113*, and *MYB114* was not altered (Tables S1 and S2). Therefore, it can be deduced that the TTG1-dependent MBW complex promotes *TT8* expression probably independent of *GL2,* or the simulative effect of the TTG1-dependent MBW complex and/or other downregulated regulatory genes is stronger than the inhibitory effect of GL2 on the *TT8* expression, in the young shoots and expanding true leaves. Similarly, the simulative effect of the TTG1-dependent MBW complex and/or other downregulated regulatory genes is comparable to the inhibitory effect of GL2 on the expression of *PAP1*, *PAP2*, *MYB113*, and *MYB114*. These interesting questions need further investigation. Genetic analysis showed that the double mutant of *ttg2-1 gl2-1* has more defective trichome development than in either single mutant, and TTG2 positively regulates *GL2* [57]. Thus, it is possible that the reduced expression of *GL2* is caused by the lower expression of *TTG2*, which would be in accordance with *GL2* being indirectly regulated by the TTG1-dependent MBW complex (Figure S3). The bHLH transcription factor MYC2 as the central mediator of the phytohormone jasmonic acid (JA) signaling positively regulates trichome initiation and flavonoid accumulation [50,51,52,53]. The previous study indicated that all the three phytohormones inclusive of JA, gibberellin A3 (GA3), and cytokinin (6-benzylaminopurine) stimulate trichome initiation not through regulating the *TTG1* expression, and whereas both JA and 6-benzylaminopurine promote anthocyanin production, GA3 does not [62]. The expression of JA biosynthetic genes was not altered in the *ttg1-13* young shoots and expanding true leaves (Tables S1 and S2), indicating that the TTG1-dependent MBW complex and JA function in an independent manner in inducing the expression of *MYC2*, thus promoting trichome formation and flavonoid accumulation. Gibberellin 2-β-DIOXYGENASE6 (GA2OX6) encoding a GA 2-oxidase functions in converting bioactive gibberellins and their precursors into inactive forms, thus reducing endogenous bioactive gibberellins [83]. CYTOKININ DEHYDROGENASE4 (CKX4) encodes a cytokinin oxidase that is responsible for the cytokinin accumulation [84]. The expression levels of *GA2OX6* and *CKX4* were significantly decreased in the *ttg1-13* young shoots and expanding true leaves (Table S1), suggesting that the TTG1-dependent MBW complex promotes trichome formation, and flavonoid accumulation might rely on cytokinin, but not gibberellins.

We demonstrated that the TTG1-dependent MBW complex directly promoted the expression of the regulatory gene *BLT* (Figure 3 and Figure 4) and indirectly activated the expression of the regulatory genes *SPL8*, *ETC1*, *HDG11*, *CPC*, *MYB106*, *MYB5*, *SIM*, and *MYB23*, and the structural gene *SVB* during trichome formation in the young shoots and expanding true leaves (Figure S3). BLT encodes a key regulator of trichome branching, and its mutation results in the formation of branchless trichomes with blunt tips [54,55]. SPL8 positively regulates the trichome number on flower sepals [48]. ETC1 acts in concert with CPC to repress the trichome cell fate in the shoot epidermis [49]. SVB is positively correlated with trichome size, and its mutant exhibits trichome branches of variable length and number [65]. HDG11 encodes a homeodomain leucine zipper transcription factor like GL2 and displays a positive role in trichome differentiation [56]. MYB106 functions as an activator in trichome differentiation [58], and a repressor in trichome branching [59]. Loss of functions of MYB5 and MYB23 display increased numbers of small and two-branched trichomes, respectively [60,61]. Further genetic analysis indicated that MYB5 and MYB23 are partially redundant in repressing trichome branching [60]. *MYB23* is directly activated by GL2 during trichome formation [56], suggesting that the TTG1-dependent MBW complex promotes the expression of *MYB23* possibly via the direct activation of *GL2*. SIM encodes a member of plant-specific cyclin-dependent kinase inhibitors, and its mutation results in multicellular trichomes [63,64]. Considering that there is no trichome present in the *ttg1-13* young shoots and expanding true leaves (Figure S1), it is plausible to assume that the TTG1-dependent MBW complex promotes trichome formation first by activating the expression of the genes that induce trichome initiation and differentiation, including *TTG2*, *GL2*, *MYC2*, *SPL8*, *HDG11*, and *MYB106*, and then by promoting the other genes that positively regulates branching, size, and cell number, including *BLT*, *MYB5*, *SIM*, *MYB23*, and *SVB*, in the young shoots and expanding true leaves. Further, the TTG1-dependent MBW complex might be independent of *ETC1* and *CPC* or might be due to the fact that the negative influence of the two genes is noncompetitive with the positive effect of other genes during trichome formation in the young shoots and expanding true leaves.

On the other hand, we proved that the TTG1-dependent MBW complex directly promoted the expression of the regulatory gene *TT8* and the structural genes *F3’H*, *DFR*, *ANS*, *UGT79B1*, *UGT75C1*, and *5MAT* (Figure 3 and Figure 4), and indirectly activated the expression of the regulatory gene *ANL2* and the structural genes *PAL4*, *DMR6*, *FLS3*, and *GSTF12* during flavonoid biosynthesis in the young shoots and expanding true leaves (Figure S3). PAL comprises four isoforms of PAL1, PAL2, PAL3, and PAL4 that convert phenylalanine into *trans*-cinnamic acid, which is the first step in the flavonoid biosynthetic pathway in *A. thaliana* [70,71]. F3’H encoding a cytochrome P450 monooxygenase catalyzes the formation of eriodictyol and dihydroquercetin from naringenin and dihydrokaempferol, respectively [70,72]. DMR6 is a flavone synthase I enzyme that catalyzes the conversion of the flavanones into flavones [73]. FLS acts as the first committed enzyme for flavonol biosynthesis [85,86], and there are five *FLS* genes in the *A. thaliana* genome, among which FLS3 exhibits the FLS activity, promoting flavonol accumulation [74]. DFR catalyzes the formation of leucoanthocyanidins from dihydroflavonols [2,75]. ANS encoding a 2-oxoglutarate-dependent dioxygenase catalyzes the conversion of leucoanthocyanidins into 3-OH-anthocyanins [76,77]. UGT79B1 is involved in the glycosylation of anthocyanins at the C-5 position, and its knockout mutant contains a drastically decreased content of anthocyanins [70,78]. Another UDP-glucose: UGT75C1 is involved in the malonylation of anthocyanins, and its mutation results in the complete loss of anthocyanin 5-*O*-glucosides [79]. 5MAT is specific for malonyl–CoA and for anthocyanins with 5-*O*-glucosylation and accelerates the accumulation of malonylated anthocyanins [80]. GSTF12, a member of GST-like proteins, is involved in transport and promotes anthocyanin accumulation [81,82]. ANL2, as a member of homeodomain proteins, positively regulates anthocyanin accumulation in shoot cells [67]. These suggested that the TTG1-dependent MBW complex promotes flavonoid biosynthesis directly or indirectly through a series of regulatory and structural genes, and TTG1-mediated flavonoid biosynthesis might be independent of *GL2* or might be due to the fact that the negative influence of GL2 is noncompetitive with the positive effect of other genes, in the young shoots and expanding true leaves.

Previous studies indicated that the TTG1-dependent MBW complex has no obvious effect on *MYB5* expression in seeds [60] and directly activates *BAN* expression in siliques [46]. Here, we found that *MYB5* and *BAN* were indirectly activated and not regulated, respectively, in the *ttg1-13* young shoots and expanding true leaves (Figure S3; Tables S1 and S2), indicating that the TTG1-dependent MBW complex regulates gene expression is tissue-dependent during trichome formation and flavonoid accumulation. It is worth noting that 8 and 7 downregulated genes involved in flavonoid accumulation were directly and indirectly regulated by the TTG1-dependent MBW complex, whereas 2 and 11 downregulated genes related to trichome formation were directly and indirectly regulated by the TTG1-dependent MBW complex in the young shoots and expanding true leaves (Figure 3 and Figure 4, and Figure S3; Table 1 and Table 2, Tables S1 and S2), suggesting that the TTG1-dependent MBW complex is likely to be a key direct and indirect transcription factor in regulating flavonoid accumulation and trichome formation, respectively.

In the present study, we report on direct targets of the TTG1-dependent MBW complex, revealing that the TTG1-dependent MBW complex functions primarily or exclusively as a transcriptional direct and indirect activator in the regulation of trichome formation and flavonoid accumulation, respectively, in the young shoots and expanding true leaves of *A. thaliana*.

## 4. Materials and Methods

### 4.1. Plant Material and Growth Condition

The *A. thaliana* ecotype Columbia (Col-0) was used as the wild type control. The *ttg1-13* mutant was utilized in our previous study [14]. The growth condition of *A. thaliana* plants has been described previously [14].

### 4.2. Generation of Transgenic Plants

The construct of *35S:TTG1-GR*, which was created in our previous study [7], was transformed into the *ttg1-13* mutant via floral dip [87]. The *ttg1-13 35S:TTG1-GR* transgenic plants were selected by Basta on soil and also verified by DNA analysis until T3 homozygous transgenic progeny was generated.

### 4.3. RNA Sequencing (RNA-seq) and Data Analyses

The samples of young shoots and expanding true leaves used for RNA-seq analysis were carefully harvested from Col-0 and *ttg1-13* plants at 20 DAG following the previously reported method [88]. Three independent biological replicates from three different plantings were performed in the RNA-seq experiment. The RNA-seq and data analysis were carried out through the Gene Denovo service (http://www.genedenovo.com/) following the standard protocol (http://www.genedenovo.com/product/41.html): (1) The quality and quantity of the isolated RNA samples were assessed by Nanodrop 2000 Spectrophotometer (Thermo, Wilmington, NC, USA) and Agilent 2100 Bioanalyzer (Agilent, Böblingen, Germany), (2) removement of the possible DNA contamination with DNase I, (3) mRNA enrichment and fragmentation, (4) sequencing adaptor ligation and PCR amplification, and (5) assessment on quality and quantity of the sample library. Finally, the cDNA library products were utilized for sequenced analysis via the Illumina HiSeq™ 2000. Transcript abundance was calculated as RPKM (reads per Kb per million reads) [89]. RPKM values presenting as “0” were artificially set to “0.001” for subsequent analysis. Comparisons of RPKM between treatments (WT (Col-0) vs. *ttg1-13*) were performed for each Unigene. The DEGs were functionally classified using the biological process category of Arabidopsis Gene Ontology (http://www.geneontology.org). A differential expression analysis of the two treatments was conducted using the DESeq R package (1.10.1). The resulting *p*-values were adjusted using Benjamini and Hochberg’s approach for controlling the *false discovery rate* (*FDR*). The DEGs with |log_2_ ratios| ≥ 1 and *FDR* ≤ 0.05 are functionally categorized and listed in Tables S1 and S2.

### 4.4. Gene Expression Analysis

The tissues of young shoots and expanding true leaves used for gene expression analysis are the same as those in the RNA-seq experiment. They were collected from eight individual plants at 20 DAG, and three independent biological replicates were performed for gene expression analysis. Total RNA extraction and first-strand cDNA synthesis were conducted according to the method previously described [90]. The SYBR Green Master Mix (TaKaRa Bio, Dalian, China) was utilized for qRT-PCR reaction with QuantStudio^TM^ 7 Flex Real-Time PCR System (Life technologies, Carlsbad, CA, USA), and the relative gene expression level was calculated as reported previously [91]. The house-keeping gene *ACTIN7* was used as the internal control. Primers used for qRT-PCR analysis are listed in Table S3.

### 4.5. GR Induction

For the induction of *TTG1*:*GR*, young shoots and expanding true leaves were harvested from 8 individual plants at 20 DAG 1 and 3 h after different treatments of mock, DEX, CYC, and DEX plus CYC. The concentrations of DEX and CYC used here were 10 and 5 μM, respectively.

### 4.6. ChIP Assay

The tissues of young shoots and expanding true leaves at 20 DAG used for ChIP assay are the same as those in the RNA-seq experiment. The *ttg1-13 35S:TTG1-GR* plants after germination were treated for 10 days with mock and 10 μM DEX every other day, after which they were used for the ChIP experiment. The ChIP assay was carried out as described previously [7]. In brief, 3 g of young shoots and expanding true leaves were fixed with 37% formaldehyde. After nuclear protein–DNA extraction and sonication, immunoprecipitation was conducted using a GR antibody coupled to magnetic beads. The relative enrichment of each fragment was detected by qRT-PCR, and the ChIP experiment was performed in three biological replicates. Primers used for ChIP assay are listed in Table S3.

### 4.7. Statistical Analysis

Completely randomized block designs were utilized in three biological replicates. Data were analyzed with use of the SPSS statistical package (version 8.0). The two-tailed paired Student’s *t*-test was used to analyze gene expression. *p* values ≤ 0.05 indicated a statistically significant difference.

## Figures and Tables

**Table 1:** Differentially expressed genes (DEGs) contributing to trichome formation in the young shoots and expanding true leaves of *ttg1-13* plants at 20 days after germination. DEGs with |log_2_ ratios| ≥ 1.00, and only GO Slim IDs with *FDR* ≤ 0.05, are listed here.

Differentially Expressed Genes	log_2_ Ratios	Functions	References
*SPL8* (AT1G02065)	−2.36	Promoting trichome formation	[48]
*ETC1* (AT1G01380)	−3.73	Repressing trichome formation	[49]
*MYC2* (AT1G32640)	−1.27	Promoting trichome initiation	[50,51,52,53]
*BLT* (AT1G64690)	−2.10	Promoting trichome branching	[54,55]
*HDG11* (AT1G73360)	−1.78	Promoting trichome differentiation	[56]
*GL2* (AT1G79840)	−6.33	Promoting trichome differentiation	[22,23,24]
*TTG2* (AT2G37260)	−7.89	Promoting trichome formation	[25,57]
*CPC* (AT2G46410)	−4.09	Repressing trichome formation	[49]
*MYB106* (AT3G01140)	−2.82	Promoting trichome differentiation and repressing trichome branching	[58,59]
*MYB5* (AT3G13540)	−7.07	Repressing trichome branching	[60,61]
*TT8* (AT4G09820)	−7.14	Promoting flavonoid accumulation	[62]
*SIM* (AT5G04470)	−1.94	Associated with trichome development	[63,64]
*MYB23* (AT5G40330)	−7.13	Repressing trichome branching	[60,61]
*SVB* (AT1G56580)	−1.43	Associated with trichome size and branching	[65]

**Table 2:** Differentially expressed genes (DEGs) contributing to flavonoid biosynthesis in the young shoots and expanding true leaves of *ttg1-13* plants at 20 days after germination. DEGs with |log_2_ ratios| ≥ 1.00, and only GO Slim IDs with *FDR* ≤ 0.05, are listed here.

Differentially Expressed Genes	log_2_ Ratios	Functions	References
*MYC2* (AT1G32640)	−1.27	Promoting flavonoid accumulation	[50,51,52,53]
*GL2* (AT1G79840)	−6.33	Inhibiting anthocyanin accumulation	[66]
*TTG2* (AT2G37260)	−7.89	Promoting flavonoid accumulation	[25,57]
*ANL2* (AT4G00730)	−1.06	Promoting anthocyanin accumulation	[67]
*TT8* (AT4G09820)	−7.14	Promoting flavonoid accumulation	[68,69]
*PAL4* (AT3G10340)	−3.72	Converting phenylalanine into *trans*-cinnamic acid	[70,71]
*F3’H* (AT5G07990)	−4.08	Converting naringenin and dihydrokaempferol into eriodictyol and dihydroquercetin, respectively	[70,72]
*DMR6* (AT5G24530)	−9.50	Converting flavanones into flavones	[73]
*FLS3* (AT5G63590)	−7.55	Promoting flavonol accumulation	[74]
*DFR* (AT5G42800)	−10.85	Converting dihydroflavonols into leucoanthocyanidins	[2,75]
*ANS* (AT4G22880)	−10.68	Converting leucoanthocyanidins into 3-OH-anthocyanins	[76,77]
*UGT79B1* (AT5G54060)	−8.29	Involved in the glycosylation of anthocyanins	[70,78]
*UGT75C1* (AT4G14090)	−4.09	Involved in the malonylation of anthocyanins	[79]
*5MAT* (AT3G29590)	−11.05	Involved in the accumulation of malonylated anthocyanins	[80]
*GSTF12* (AT5G17220)	−3.73	Involved in transport and accumulation of both anthocyanins and proanthocyanidins	[81,82]

## Supplementary Materials

Supplementary materials can be found at https://www.mdpi.com/1422-0067/20/20/5014/s1. Supplementary Figure S1. The schematic diagram shows tissues from the wild type (Col-0) and *ttg1-13* plants at 20 days after germination harvested for the RNA-seq experiment. The yellow crosses indicate that the whole leaves, including blades and petioles, were removed completely, whereas the red ellipses indicate that the tissues, including young shoots and expanding true leaves, were collected for RNA-seq analysis. Supplementary Figure S2. Generation of a *ttg1-13 35S:TTG1-GR* transgenic line containing the biologically active TTG1-GR fusion, and the tissues of young shoots and expanding true leaves indicated by the red ellipses were collected for GR induction and ChIP analyses. Individual seedlings of *ttg1-13 35S:TTG1-GR* plants were mock-treated (Mock, left) or treated with 10 μM dexamethasone (Dex, right) every other day after germination. Mock plants generate leaves without flavonoids and trichomes as *ttg1-13*, whereas DEX treatment rescues these phenotypes. Supplementary Figure S3. The genes involved in flavonoid biosynthesis and trichome formation are not immediate targets of transcriptional regulation by TTG1 in the young shoots and expanding true leaves. The *ttg1-13 35S:TTG1-GR* young shoots and expanding true leaves at 20 days after germination were mock-treated (Mock) or treated with 10 μM dexamethasone (DEX), 5 μM cycloheximide (CYC), or 10 μM DEX plus 5 μM CYC (DEX + CYC). The expression of these genes was determined after 1 or 3 h of treatment using qRT-PCR analyses. The house-keeping gene *ACTIN7* was used as the internal control. The expression level of each gene was first calculated relative to *ACTIN7*, and its expression levels in both Mock and CYC treatments were set to 1. Asterisks indicate significant differences in gene expression in DEX-treated samples compared with their respective controls (two-tailed paired Student’s *t* test, *p* ≤ 0.05). Values are means ± SD (*n* = 3). Error bars denote SD. Supplementary Table S1. A list of genes expressed at lower levels (‘downregulated’) in the young shoots and expanding true leaves of *ttg1-13* plants than in wild type rosette leaves at 20 days after germination. Supplementary Table S2. A list of genes expressed at higher levels (‘upregulated’) in the young shoots and expanding true leaves of *ttg1-13* plants than in wild type rosette leaves at 20 days after germination. Supplementary Table S3. Primers used in the present study.
